# Implementation of active methodologies: a six-phase instructional design model from a neuroeducational perspective

**DOI:** 10.3389/fnhum.2026.1820271

**Published:** 2026-06-24

**Authors:** Jessica Goset-Poblete, Rubén Rodríguez-Amador, Ricardo Ramírez-Barrantes

**Affiliations:** 1 Neuroeducation and Well-being Laboratory, Faculty of Medicine, Andrés Bello University, Viña del Mar, Chile; 2 Directorate of Educational Innovation and Learning Assessment, Faculty of Education and Humanities, Andrés Bello University, Santiago, Chile

**Keywords:** active learning, instructional design model, neuroeducation, qualitative process evaluation, teacher experience

## Abstract

**Introduction:**

Active methodologies are widely recognized for their potential to enhance learning in higher education. From a neuroeducational perspective, these approaches involve motivational and self-regulatory processes that are theoretically associated with dopaminergic and serotonergic functioning. This study examines the implementation of these methodologies, proposing a six-phase instructional design model, interpreted through this neuroeducational framework.

**Methods:**

A qualitative, descriptive, and exploratory study was conducted with 10 university teachers at Andrés Bello University (UNAB). Data were collected through focus groups, semi-structured interviews, and field notes, and analyzed using an iterative thematic analysis with triangulation across different data collection phases.

**Results:**

The results describe a progressive process moving from initial uncertainty to an increasing mastery of the instructional design. Across the phases, categories such as uncertainty, engagement with the challenge, close mentoring, adjustment and reconstruction, support regulation, teacher transfer, effort-reward balance, intrinsic motivation, and personal impact emerged, highlighting the experiential and evolving nature of implementation.

**Discussion:**

The findings suggest that implementing active methodologies is a dynamic process of motivation, regulation, and adaptation over time, which aligns with theoretical models of dopaminergic–serotonergic co-regulation. Ultimately, this study introduces a process-oriented framework that integrates pedagogical design and teacher experience, advancing to understand the implementation of active methodologies in complex educational contexts.

## 1 Introduction

Although active methodologies (AMs) have been part of pedagogical discourse since John Dewey’s contributions in 1916 (as cited in Baraldi, 2021), their widespread adoption occurred during the COVID-19 pandemic. In this isolation context, the urgent need to maintain educational access, conbined with rapid technological development, drove the creation of interactive materials aimed at promoting student autonomy (Antón-Sancho et al., 2022; Oliveira et al., 2021). Furthermore, their positive impact on well-being—by promoting dialogue, the mediating role of the teacher, and respect for diversity—solidifies AMs as a cornerstone of pedagogical innovations aimed at curricular humanization (Ribeiro-Silva et al., 2022).

However, disparities in educational contexts, infrastructure, and teacher training—coupled with rapid technological advancement—have led to multiple adaptations of AMs. Rather than clarifying implementation, these variations have contributed to conceptual and methodological confusion (Crous et al., 2024). Additionally, an instrumental emphasis on emerging digital resources has favored superficial approaches, fostering teacher frustration due to the absence of expected results (Amemasor et al., 2025; Wu et al., 2026).

From a neuroscientific perspective, AMs are grounded in the premise that learning is an intrinsically active process, supported by brain plasticity. Environmental experience and interaction induce the reorganization of pre-existing neural networks, thereby generating new adaptive responses (Magee and Grienberger, 2020). The richness, modification, and complexity of these networks determine the depth and significance of learning (Posner and Rothbart, 2023).

These networks involve both local synaptic connections and the functional integration of multiple brain structures. For instance, the prefrontal cortex and the limbic system are structurally interconnected, enabling a continuous synergy between cognition and emotion (Herd et al., 2021). Consequently, higher-order processes—such as decision-making, self-regulation, and planning—simultaneously involve motivation, engagement, and satisfaction (Cando et al., 2025; Luria et al., 2021). Within these integrated networks, mesolimbic and mesocortical dopaminergic pathway play a central role (Luria et al., 2021). The mesolimbic pathway, activated by goal anticipation and attainment, reinforces behavior via dopamine release to mediate reward and motivation (Bech et al., 2023; Oettl et al., 2020). This process is dynamically modulated by the reward prediction error mechanism, which influences intrinsic motivation and behavioral adjustment (Deng et al., 2023): when outcome exceed expectations, a dopaminergic peak; conversely, when expectations are unmet, a dopaminergic decline promotes cognitive reevaluation and adaptation. This bidirectional mechanism provides pedagogical validation for incorporating challenges, progressive goals, and problem-solving, aligning with the principle of “learning by doing”. Parallelly, the mesocortical pathway contributes to cognitive stability and flexibility. Here, the complementary action of D1 and D2 dopaminergic receptors regulates information input and working memory manipulation, minimizing internal and external interference (Ott and Nieder, 2019). This pathway also modulates attention, motivation, and mental fatigue (Kok, 2022), thereby adjusting behavioral learning rates and facilitating adaptation to novel challenges (Coddington et al., 2023).

However, an exclusive focus on dopamine has led to reductionist interpretations in pedagogical implementation. Constant stimulation through superficial rewards or overdesigned activities fails to guarantee optimal learning, often induce stress and cognitive overload (Evans et al., 2024). Because effective learning regulation relies on a complex interplay among neuromodulatory systems, the serotonergic system exerts a critical balancing role key modulatory role. Beyon its established function in emotional regulation, serotonin modulates dopaminergic pathways to influence goal-directed motivation, reward valuation, and punishment avoidance (Courtiol et al., 2021). Specifically, serotonergic projections from the raphe nuclei to the prefrontal cortex—including the medial orbitofrontal cortex—are involved in option evaluation, behavioral inhibition and adaptation to changing contexts (Fischer and Ullsperger, 2017). Furthermore, these projections help maintain dopaminergic activity within an optimal range to maximize cognitive efficiency (Ott and Nieder, 2019). While dopamine is predominantly released in rapid phasic peaks associated with surprise or reward anticipation, serotonin exhibits tonic and phasic patterns, frequently interacting with glutamate (Liu et al., 2020). Serotonin release is uniquely associated with environments characterized by safety, social acceptance, rhythmic motor activity and contact with nature (Kikuchi and Noriuchi, 2020; Li et al., 2022).

Thus, while dopamine drives goal-oriented approach behavior, serotonin provides the evaluative control and emotional regulation necessary for an adaptive learning trajectory (Liebana et al., 2025). The dynamic co-regulation between the dopaminergic and serotonergic systems allows balance between motivation and reflection, challenge and stress regulation, promoting deep learning under conditions of well-being. From this perspective, the pedagogical challenge of AMs shifts from maximizing external stimuli to designing integrated experiences that foster such neurobiological co-regulation.

Although some studies have reported direct brain-level data (Smith et al., 2025), the difficulty of directly recording neurotransmitters in educational contexts has led neuroeducational models to focus on behavioral indicators (Elouafi et al., 2021). Giving the neurochemical bases underpinning human reactions, qualitative observation and behavioral analysis enable rigorous inferences regarding these underlying processes (Luria et al., 2021). However, considering the complexity and multifactorial nature of human behavior, current neuroeducational framework present a gap in addressing the temporal dimension of these models.

To brigde this gap, the present study proposes a model for designing educational experiences implemented over six months, aimed at promoting learning processes aligned with the dopaminergic and serotonergic co-regulation systems. Regardless of the specific active methodology employed, the model focuses on creating integrated experiences that optimize cognitive efficiency while promoting well-being, intrinsic motivation, and sustained self-regulation over time. Rather than introducing a new active methodology, this study aim to answer the research question: How do teachers experience a six-phase neuroeducational design model for the implementation of active methodologies? Its novelty lies in (i) the identification of phase-based experiential dynamics, (ii) the articulation of these dynamics across a six-phase temporal model, and (iii) the integration of these processes through a neuroeducational interpretative framework. This approach bridges pedagogical design and teacher experience in real institutional contexts.

## 2 Methods

### 2.1 Study design

This pilot study employed a qualitative, exploratory, and descriptive design aimed at understanding the phenomenon in depth from the participants’ perspective. Specifically, an iterative thematic analysis was utilized to understand how teachers experienced the implementation of active methodology within an educational innovation framework, structured across a six-phase neuroeducational model. This approach is relevant when the objective is to understand the dynamics of change, its implementation conditions, and the mechanisms that facilitate or hinder its development in real contexts, in line with contemporary frameworks for the study of complex interventions, sensitive to their evolution and contextual adaptation (Moore et al., 2015; Skivington et al., 2021).

Methodologically, the study is positioned within a qualitative tradition designed to understand lived experiences, meanings, and interpretation processes in context, rather than in testing causal relationships. The analysis was thus conducted as an iterative, reflective and progressive process, consistent with current frameworks that view qualitative research as an analytical construction sustained through successive decisions of coding, comparison, and interpretation (Dahal, 2025; Hoff and Strømme, 2025). Crucially, while neuroscientific literature informed the conceptual framework of the model and guided the theoretical discussion; it did not constitute an empirical outcome measured in this study.

### 2.2 Participants

The study was conducted with 10 teachers from different disciplines at Andrés Bello University (UNAB), who participated as academic leaders in educational innovation for the Digital Update of the Autonomous Study Guide (ADIGEA) within the Faculty of Medicine. The inclusion criteria required that the faculty member responsible for the disciplinary academic responsible for the specific course participates actively in the implementation process. Conversely, the exclusion criteria were defined in relation to contractual conditions that would prevent sustained participation throughout the six phases of the model (Table 1).

**Table 1:** Description of the teachers participating in the study.

Teachers	Discipline	Age	Gender	Teacher’s experience in years	University campus
1	Obstetrics	42	Female	5	Viña del Mar
2	Obstetrics	53	Female	10	Concepción
3	Medicine	45	Male	15	Santiago
4	Medicine	58	Female	3	Santiago
5	Medical Technology	43	Female	10	Viña del Mar
6	Medical Technology	44	Male	8	Viña del Mar
7	Nutrition and dietetics	54	Female	16	Santiago
8	Nutrition and dietetics	45	Female	10	Concepción
9	Chemistry and Pharmacy	55	Female	17	Viña del Mar
10	Chemistry and Pharmacy	53	Male	2	Viña del Mar

The sampling strategy was designed to achieve heterogeneity regarding teaching experience, gender, and university campus. Given the limited sample and institutional specificity, however, the findings remain exploratory and context-specific rather than broadly generalizable. The underlying instructional model comprised six phases: preparation, immersion, transfer, implementation, evaluation, and dissemination. This structure guided both the intervention and the data collection processes over a six-months period.

### 2.3 Description of the six-phase model

The six-phase model was conceived as a structured process based on the theory of reward system co-regulation, selecting activities related to support, immediate and delayed rewards, social recognition, a safe environment, contact with nature, and body–mind activities.

#### 2.3.1 Preparation phase

The challenge of digital updating for the self-study guide begins with the search and classification of the educational resources that have previously been used by teachers in the subject. Next, they independently assess whether these resources are sufficient, need to be modified, or new ones should be created. In addition, they must identify the need for guidance on modifying or developing digital resources.

The objective in this phase is to present the initial challenge in a simple and broad way, to introduce productive uncertainty and activate search behavior, modulated by the certainty of support and the commitment of the advisory team.

#### 2.3.2 Immersion phase

The immersion phase is aimed at solving micro challenges corresponding to the requirements previously identified by participants in the preparation phase. For this, a team of educational and technological advisors is available to facilitate the learning process.

This phase takes place in a physical space outside the academic environment, with scheduled breaks, during an 8-h session. These breaks, lasting 20 min each, were designed based on the body–mind connection and contact with nature, and they focus on breathing exercises, organic movement, balance, and walks through the forest. This immersive experience was designed for participants to achieve a concrete and meaningful accomplishment in their teaching role, feeling recognized and part of a work team in a supportive environment.

#### 2.3.3 Transfer phase

The transfer phase aims to reinforce the knowledge acquired by explaining it to peers. To achieve this, online meetings were arranged to share project progress, explain critical points, gather concerns, and guide the team’s integrated progress. Support is also present in this phase, with the advisor being responsible for starting the session and then handing over the leadership of the meeting to the responsible teacher, backing them up with acquired knowledge, management of the teaching team, and institutional support.

#### 2.3.4 Implementation phase

The teacher leads the launch of the project, ensuring the proper integration of the entire academic team and making decisions based on the knowledge acquired, which can generate high levels of stress. This phase is characterized as cognitively and emotionally complex. It was based on motivation, self-regulation, and delayed reward; in fact, to modulate it, a communication strategy was designed that provides quick access to advice.

#### 2.3.5 Evaluation phase

The evaluation phase was cyclical in relation to implementation; however, there were two assessments designed to promote the activation of the reward system. The first is related to recognizing the group and individual experience of early implementation and analyzing the partial quantitative and qualitative results of the implementation. The second corresponds to the analysis of the final mixed data and group reflection to define areas for improvement and seek the teaching strategy to achieve them. Guidance in this phase mediates the reflective process to identify changes and facilitate awareness of achievement.

#### 2.3.6 Dissemination phase

It consisted of allowing the teacher to present the results of the project implementation orally in a setting outside their usual environment, with high professional and social recognition, such as a conference related to innovation. For this, the teacher was once again accompanied, generating collaborative instances with their pair work and the pedagogical advisor to obtain feedback on the work to be presented. It is important to highlight that, as in the immersion phase, the experience was designed to enhance belonging, achievement, support, sense of community, and recognition. This phase is the culmination of the process and represents a significant cognitive and emotional challenge.

### 2.4 Data collection procedures and techniques

The data were collected at critical moments over a six-month period, using complementary qualitative techniques. The strategy combined focus groups, semi-structured interviews, and field notes from one of the researchers (Table 2). This articulated use of techniques is consistent with qualitative approaches aimed at capturing both individual narratives and processes of collective sense-making around implementation experiences, and it met widely recognized criteria for the design and reporting of qualitative studies based on interviews and focus groups (O’Brien et al., 2014; Tong et al., 2007). Moreover, the combined use and timing of data collection proved relevant in applied studies seeking to understand complex developmental processes, thereby favoring a more situated and robust interpretation of the phenomenon under study (Fredkove et al., 2024; López et al., 2025).

**Table 2:** Research objectives, instruments and types of inference.

Research objectives	Instruments	Types of inference
Analyse the experience of teachers in the preparation and immersion phases.	Focus groups	What they think about support, sense of achievement and the work environment. Likewise, what they think about the challenge to be met.
Analyse the experience of teachers during the phases of transfer and implementation.	Semi-structured interview	What they think about pair works transfer, the challenge to meet again, the support.
Analyse the experience of teachers during the evaluation and dissemination phases.	Focus groups	What they think about achievement, support, pair work relationships and self-efficacy.

During the preparation and immersion phases, an initial focus group was held, divided into two subgroups at the end of the working session, in order to capture the initial participant perceptions of the challenge, the role of support, and the sense of achievement associated with the experience. For this purpose, a set of questions was designed, including: How did you experience this preparation and immersion? How did you feel when facing the challenge posed? Which aspects did you find most demanding or uncertain during the preparation and immersion phases? How did you perceive the support received during the process? In what way did the advisory team’s support facilitate or hinder your progress? What type of support was most relevant for you during these phases? What sense of achievement does this experience leave you with? At what point did you feel that you were making progress or had achieved something significant? What concrete learning outcomes or results do you consider you have attained by the end of the phases?

In the transfer and implementation phases, semi-structured interviews were conducted by disciplinary groups, which allowed for a deeper understanding of aspects related to implementation decisions, redesign adjustments, dependence on key actors, tensions, and sustainability conditions. In this regard, questions such as the following were used: How was the proposal transferred to other spaces, courses, campuses, or teachers? Had they already put it into practice, or was it still in the process of transfer? What exactly happened when they began to transfer it? What decisions were necessary to implement the proposal under real conditions? How did they organize the implementation? Which elements of the activity design helped the proposal work best? What difficulties or obstacles arose during implementation? What happened during that process, and what tensions did you have to resolve? What type of obstacles were most relevant: pedagogical, technological, organizational, or communicative? Why do you consider that the proposal worked or did not work in practice? How do you explain the success of the implementation in the locations or contexts where it worked best? What factors were decisive in maintaining its functioning?

In the evaluation and dissemination phases, focus group were conducted to examinate learning outcomes, closing reflections, and the perceived effects of the process as a whole. In this final phase, questions were designed such as: What do you consider were the main learnings this process left you with? What effects did this experience have at the conclusion of the process on your way of understanding or practicing teaching? What changes do you recognize in yourselves after going through all the stages of the process? Which aspects of the process do you consider to have been the most valuable at the end of the experience? Which elements allowed you to reach a satisfactory conclusion, and which remained unresolved or were tense? How do you evaluate, in retrospect, the entire journey experienced from preparation to dissemination? What impact did the stage of dissemination or socialization of the work have? What meaning did sharing, showing, or projecting what you developed have for you?

In addition to the focus groups and the semi-structured interviews, field notes prepared by one of the researchers during the course of the process were considered. These notes were complementary in nature and were used to contextualize interactions, record relevant situational elements, and support the organization of the corpus according to the different phases of the instructional model. Within the framework of iterative thematic analysis, reviewing them alongside the transcripts confirmed the assignment of the narratives to each phase, clarified the implicit meanings, and deepened the interpretation of the teachers’ lived experience, thus strengthening the rigor and coherence of the analysis.

All formal sessions lasted approximately 45 min and were recorded in audio and/or video. The material was transcribed and managed using Dedoose software to support data organization, coding, and traceability of the analytical process. The use of the software was limited to technical support in corpus management, without replacing the interpretation developed by the research team, in line with strict recommendations for transparency in qualitative research (Tong et al., 2007; O’Brien et al., 2014; López et al., 2025).

### 2.5 Data analysis

The information collected was analyzed through an iterative thematic analysis, aimed at identifying patterns of meaning in participants’ accounts throughout the different phases of the process. The study categories were derived from a qualitative thematic analysis process supported by two complementary data collection techniques: an initial focus group, centered on the preparation and immersion phases (FPEIN); a semi-structured interview, aimed at the transfer and implementation phases (FTEIM); and a second focus group, aligned with the evaluation and dissemination phases (FEVYD). In this way, these techniques provided convergent and complementary information for the identification of categories such as uncertainty (IN), appropriation the challenge (AD), close mentoring (AC), adjustment and reconstruction (AR), support regulation (RA), teacher transfer (TD), effort-reward balance (BER), intrinsic motivation (MI) and personal impact (IP) (Hoff and Strømme, 2025; O’Brien et al., 2014).

In the first stage, open coding of the focus group corresponding to the preparation and immersion phases was carried out without predefined categories. In this phase, significant fragments of the discourse were identified, to which provisional codes were assigned with the purpose of capturing the emerging meanings of the experience narrated by the participants. In a second stage, these initial codes were grouped, refined, and compared iteratively until broader categories were consolidated and defined operationally based on the narratives themselves. When segments emerged that were not adequately represented by the coding framework under development, new categories were generated or previously established ones were revised. This procedure allowed for maintaining the analytical flexibility necessary to account for nuances, shifts, and tensions present in the studied experience.

In order to strengthen the traceability of the analysis and preserving fidelity to participants’ accounts, working tables were created by phase and category in which the most representative statements from the corpus were gathered. Each extract was identified using analytical codes, which allowed for recognizing its phase of origin, its categorical assignment, and its location within the analysis process. From these matrices, synthetic descriptions of each category in each phase were written, initially maintaining a descriptive level close to the discourse of the participants. This procedure allowed for a first descriptive reduction of the material before moving toward a broader analytical integration of the findings.

Once the analytical categories were identified, a systematic review of the empirical corpus was carried out for the purpose of rereading the focus group corresponding to the preparation and immersion phases, the interviews related to the transfer and implementation phases, and, finally, the focus group associated with the evaluation and dissemination phases. In this third stage, the resulting set of categories was systematically applied to the full set of documents, which allowed the complete process to be analyzed within a shared framework and to identify continuities, variations, and shifts between different phases, achieving analytical saturation in the dissemination phase.

To strengthen analytical credibility, the research team kept analytical notes, documented category adjustments, and held regular discussions to refine code definitions and resolve discrepancies (O’Connor and Joffe, 2020). Additionally, one of the researchers took field notes during the development of the different phases of the process, recording contextual observations, interaction dynamics, and emerging tensions that were not always fully reflected in the transcriptions. These notes were used as a complementary input to situate the narratives in their production context, align the reading of the corpus more precisely according to the phases of the model, and deepen the interpretation of the data.

Likewise, temporal triangulation was used to contrast the consistency and coverage of the categories at different moments in the implementation process. The analysis sought to achieve sufficient depth and coherence within the available data set. However, given the limited number of participants and the fact that the study was conducted in a single institutional context, the findings should be interpreted with caution.

### 2.6 Positionality and reflexivity of the research team

The members of the research team held different positions in relation to the design, implementation, and analysis of the study. One of the researchers was directly involved in the design of the model, the support of the implementation, the observation of the process, and the production of field notes. Another researcher took the lead in generating and analyzing information through semi-structured interviews and focus groups.

This distribution of roles offered relevant analytical advantages, particularly in terms of contextual sensitivity and comprehensiveness, by articulating perspectives with different degrees of proximity to the studied process. However, it also posed potential risks of interpretive bias associated with that differential proximity. To mitigate these risks, the team maintained a continuous reflexive practice throughout the study, critically examining their positions, assumptions, and ways of involvement in the construction of the analysis (Buckley, 2021; Hill and Dao, 2021). This practice resulted in the systematic documentation of analytical decisions, the review and discussion of coding discrepancies, and the explicit differentiation between participants’ accounts, field observations, and interpretations constructed by the research team.

### 2.7 Ethical considerations

The participants gave their informed consent before joining the study. Participation was voluntary, and each participant was informed of their right to withdraw at any time without consequences. The information collected was anonymized, securely stored, and analyzed under confidentiality criteria, in accordance with current institutional regulations. Only the research team had access to the records and transcripts, which were used exclusively for research purposes. In the presentation of results, names and potentially identifiable information were removed. These measures sought to safeguard the integrity of the participants and strengthen the transparency and auditability of the study, in line with recommendations for qualitative research and its reporting. (O’Brien et al., 2014; Tong et al., 2007).

## 3 Results

The results were organized according to the phases of the process: preparation and immersion, transfer and implementation, and evaluation and dissemination. The categories emerged from the analysis of the participants’ statements and allowed for describing the lived experiences, the initial tensions, the supports and adjustments that facilitated progress, the difficulties that arose during implementation, and the perceived effects on the teaching and student areas. Table 3 summarizes the emerging categories from the analysis, their operational definitions, and a representative illustrative statement from the analyzed narratives.

**Table 3:** Emerging categories, operational definitions, and illustrative statements.

Category	Code	Operational definition	Illustrative declaration
Uncertainty	FPEIN-IN-01	Initial uncertainty about the purpose, the work path, and the criteria for achieving change.	*“I was in a somewhat anxious state… this of not… where is it going? In this change…”*
Appropriation of the challenge	FPEIN-AD-01	Adoption of the challenge through guided action: learning by doing and mastering the minimum viable elements to operate the design.	*“The CANVAS cost me a lot… I realized that I did not know anything about the platform.”*
Close advisory	FPEIN-AC-01	Situated and continuous mediation that supports pedagogical and technical decisions during the process.	*“It was a very equal-to-equal job… that facilitates communication… and through communication you can reach a result”*
Adjustment and reconstruction	FPEIN-AR-01	Iteration of the teaching design to organize, calibrate, and give coherence to the resources, activities, and sequences.	*“At first you wanted to do everything… over-design is the same as having no design… let us calm down… one step at a time”*
Support regulation	FPEIN-RA-01	Progress conditioned by role/access dependencies and by the availability of timely support.	*“I would have needed more support from the learning technologies team… with each group… it would have flowed much more.”*
Teacher transfer	FTEIM-TD-01	Projection of innovation to replicate it and adapt it with other teachers and in new implementation contexts.	*“Responsibility… of having to transmit this to other campus… how to do it… so as not to generate resistance to change”*
Effort-reward balance	FTEIM-BER-01	Assessment of the effort and time invested versus achievements, perceived usefulness, and recognition of the work.	*“Time must undoubtedly be the greatest obstacle”*
Intrinsic motivation	FEVYD-MI-01	Sustained internal drive to persist in change out of interest, professional sense, and pedagogical purpose.	*“Now I feel like doing more… continuing to learn and sharing it”*
Personal impact	FEVYD-IP-01	Reported transformation in the experience of the teaching role (renewed perspective, practical confidence, willingness to continue)	*“I would define it with one word, growth… from a professional point of view”*

### 3.1 Preparation and immersion phase

During the preparation and immersion phase, participants described experiencing initial uncertainty. This uncertainty was related to the purpose, the work pathway, and the expected outcome. They reported initially insufficient clarity about the process, with doubts about what was expected of them and about the real and concrete possibility of obtaining a useful product. A teacher pointed out that *“I arrived with the expectation of knowing what I was coming for because I wasn’t very clear… I came in with a blank mind…” While* another participant reported that he was *“in a somewhat anxious state… this of not… where is it going? in this change…”.* This initial uncertainty was associated both with doubts about the feasibility of the product and with a feeling of tension toward the unknown. However, as the day progressed, this perception gradually shifted toward an initial sense of achievement and feasibility.

The appropriation of the challenge was configured as a practical and progressive learning process. The narratives show that the teachers began to take ownership of the challenge through exploration, trial and error, and gradual handling of tools and procedures. A participant stated*: “I struggled with CANVAS… I realized that I didn’t know anything about the platform.”* However, this appropriation was not only technical. It also involved a pedagogical shift: recognizing that innovating did not mean incorporating many activities or tools, but learning to select, prioritize, and adjust. In this sense, a teacher noted that *“at first one wants to do everything… overdesign is the same as having no design…”* Appropriation, therefore, occurred as basic mastery of the design and, at the same time, as a more refined understanding of its pedagogical purposes.

Close advising appears in this phase as a decisive condition for progress. Participants describe a horizontal, concrete, and timely support that facilitated communication and sustained decision-making. One participant expressed it by saying that *“It was a very peer-to-peer work… that makes communication easier… and through communication one can reach a result.”* Another participant reinforced this idea by pointing out that it was about *“creating, building with colleagues… everyone contributes…”* The experience was therefore lived as a process of joint construction rather than as an individual sequence of tasks.

In parallel, the adjustment and reconstruction of the design were experienced as a constitutive part of the experience. The participants describe an iterative work of reorganizing resources, activities, and sequences, aimed at calibrating the load and avoiding overdesign. A process of refinement emerges in the narratives: simplifying, balancing, and prioritizing to maintain the pedagogical meaning of the work. This reconstruction was accompanied by a growing awareness that change did not consist in doing things, but in doing them with greater coherence.

Nevertheless, the participants experienced the regulation of support as an early tension. Progress depended heavily on the presence of key roles and immediate support to maintain the flow of work. When that support was lacking, the feeling of disorientation and operational fragility increased. One participant expressed that *“I would have needed more from the learning technologies team… with each group… it would have flowed more…”*. The support was perceived as essential to the functioning of the experience.

Overall, the phase was evaluated from a predominantly positive effort-reward balance. Although the work was experienced as intense, the effort was reinterpreted as productive and valuable. A participant summarized this experience by stating*: “I felt tired… but with an appreciation… it is a different kind of tired.”* Along the same lines, intrinsic motivation appears in this phase as an incipient transformation: greater enthusiasm, perception of growth, and a different perspective on one’s own practice.

In summary, the transition from uncertainty to perceived feasibility is particularly noteworthy. The key shift lies in recognizing that innovation involves coherent selection and prioritization rather than the accumulation of activities.

### 3.2 Transfer and implementation phase

In the transfer and implementation phase, the participants described a transition from initial uncertainty to a progressive clarification of the process, now under real working conditions. Unlike the previous phase, the uncertainty was no longer focused solely on the general sense of the project, but on how to organize the implementation, sustain the workload, and transfer to heterogeneous contexts. In some cases, the main tension was expressed as communicational problems and a lack of clarity in the transmission of information. A participant put it directly by pointing out that the main difficulty was *“the transmission of information from the project management leader,”* because *“it was not clear, it was not organized,”* and, therefore, *“it was difficult.”* Along the same lines, she added that *“if from the beginning the agreements are not very clear, the truth is that things get disorganized downwards.”* In other cases, uncertainty was centered less on the initial information and more on how to maintain the transfer to other teachers and other sites without losing coherence or generating resistance.

The appropriation of the challenge in this phase was expressed mainly as a quick and functional adaptation aimed at mastering what was necessary to operate the design under real conditions. The accounts described that the teachers had to adjust their practices as the implementation progressed, often under time pressure and with limited room for maneuver. A teacher pointed out that the material *“was not completely ready”,* so she had to *“first adjust”* her lectures and then *“start to somewhat organize the dynamics in which the work was going to be carried out.”* In another case, the appropriation involved teaching other teachers to incorporate materials into the templates of their own campuses, in a process that was *“built as we went along, week by week.”* The appropriation is perceived as neither homogeneous nor linear; in some cases, it resulted in greater practical autonomy; in others, it required an adaptation by the implementation itself.

In this context, close advisory support once again became central as both pedagogical and operational support. The participants highlighted the importance of frequent coordination, peer mentoring, and the presence of figures capable of resolving critical issues. One teacher summarized this need by stating that the teams required *“accompaniment… someone asking them how they are, how it went for them”.* In other accounts, this support was described as assistance that helped organize the implementation, reduce anxiety, and maintain the continuity of the work. It was also observed that the way teams were formed affected the quality of this advice: groups built on relationships of trust and more distributed leadership progressed more quickly, while those organized under more hierarchical logics tended to ask for less help, follow instructions without consensus, and increase the anxiety of the rest of the academic team.

In this phase, constant work of adjustment and reconstruction was also required. The teachers reported successive iterations of the design to reorganize content, arrange the guiding thread, and maintain coherence between resources, activities, and timing. In several cases, the redesign was experienced as a response to the need to give more practical meaning to classroom work and to avoid both dispersion and an excess of activities. The use of support tools to measure feedback and mitigate interpersonal tensions was also highlighted, shifting the focus from individual opinion to criteria, evidence, and concrete work deadlines.

Regarding teacher transfer, the reports showed that its value increases when it is accompanied by sustained follow-up and by structures that allow continuity to be maintained. However, when projecting the innovation toward wider implementation, scaling emerged as a decisive test. A participant expressed it in terms of *“responsibility… of having to convey this to other sites… How to do it?”* to avoid producing resistance to change. Likewise, other accounts show that the transfer was not simply experienced as sending materials, but as a complex boundary between those who had participated in the design process and those who had to implement it later. In several cases, this distance generated unequal appropriations: from teachers who adjusted the material to their own style and integrated it with relative ease, to others who took it more as an external indication or a regulatory compliance.

The regulation of support emerged as one of the most critical aspects of operation. Several participants reported bottlenecks associated with dependence on key people, restricted access, limited licenses, and manual processes that made the operation fragile.

One particularly illustrative case was that of a site where, since the resource was loaded on a platform associated with only one teacher, the implementation was interrupted when that person went on medical leave because *“the resource… was lost because they could not log in.”* In another interview, it was explicitly suggested that *“it would be good for the project coordinator or manager to have access to all fields,”* precisely so as not to depend on a single account or person. In this way, the regulation of support not only refers to human presence, but also to the technical and organizational conditions of access, continuity, and timely response.

In terms of the effort-reward balance, the phase was evaluated under very clear structural limits: time, workload, and contractual conditions. The accounts link this tension to the unequal distribution of effort between design, assembly, and follow-up, as well as to the impossibility for some teachers to become fully involved due to their working conditions. In the words of one interviewee*, “the teachers are adjuncts… “There is no monetary compensation nor a contractual requirement, “*so part of the collaboration ended up being more instrumental than truly shared. Another participant directly pointed out that time was *“by far the biggest obstacle.”* Even so, the workload was not experienced solely as wear and tear. In several accounts, when the design began to show coherence and results, the effort was positively reinterpreted. Thus, intrinsic motivation appeared to be associated with the pedagogical meaning of the change, the possibility of seeing more engaged students, and the perception of organizing teaching in a more meaningful way.

Finally, the personal impact was described as a transition from initial doubts toward greater clarity and a sense of achievement. This change was associated with the recognition of gaps at the beginning, with the progressive appropriation of innovation, and with a clearer perception of its feasibility. In some cases, the implementation forced a review of deeply ingrained beliefs about the way of teaching, the control of the process, and teaching authorship itself. In others, it allowed the recognition that the effort made was positively valued as a different kind of fatigue, linked to learning and professional growth. The closing of this phase was accompanied by favorable emotions, a renewed perspective on practice, and a perception of transformation both professionally and personally related to the implementation experience.

Based on the foregoing, operationalization under pressure is particularly salient, with transfer and the regulation of support emerging as critical factors. In this context, the positive reinterpretation of effort depends on the perceived pedagogical meaning.

### 3.3 Evaluation and dissemination phase

In the evaluation and dissemination phase, the teaching experience acquired a reflective and forward-looking character at closure. Unlike the previous phases, here the narratives do not focus so much on the construction of the innovation or on the operational difficulties of its implementation, but on the reinterpretation of the lived process, on the assessment of its effects, and on the possibility of communicating it to others. In this sense, the phase functioned as a space for reflection, recognition, and openness to new academic and professional opportunities.

Close mentoring was expressed at this stage mainly through pair-work support. The participants emphasized collaboration in preparing presentations, rehearsing, correcting each other, and accompanying one another in the public presentation of their work. One participant highlighted that they were able to *“practice our presentations”* and *“make corrections to each other”* among everyone, which allowed them to strengthen both the quality of the product presented and the confidence to communicate it publicly. Along the same lines, another participant emphasized that *“this camaraderie… a space to be able to promote research, to be able to promote better teaching”* had been generated. Thus, the advisory no longer appeared only as technical or pedagogical mediation, but as a form of mutual support that consolidated the collective meaning of the process.

From the effort-reward balance perspective, the phase was marked by a predominantly positive emotional load. The participants associated the accumulated effort with professional and symbolic rewards: visibility of the work, recognition by others, validation of what had been done, and confirmation that the process had borne concrete results. A participant expressed it by pointing out that this had been *“the first experience I have of participating in a conference,”* and that this gave her a particularly significant sense of achievement. In several accounts, the reward was not limited to the fact of having completed a task, but was linked to the experience of having been part of something valued and to the possibility of communicating it in formal academic spaces.

The appropriation of the challenge was expressed in this phase as a consolidation of skills and as an expansion of professional horizons. The participants described having learned procedures, languages, and tools that previously seemed distant to them, and they recognized greater confidence in continuing to develop them. A teacher reported feeling especially satisfied upon discovering that she could understand methodological and analytical aspects that initially seemed strange to her, enthusiastically noting that she had managed to understand elements that had previously seemed completely foreign to her. In this sense, appropriation ceases to be solely operational and transforms into confidence to continue learning and producing.

Uncertainty, although already mitigated, does not disappear completely. In this phase, it manifests especially in the public exposure of the work, in the nervousness before presenting results, and in the doubt regarding the actual scope of the process carried out. However, this uncertainty is now experienced in a different way: less as disorientation and more as part of a new, challenging, and mobilizing academic experience. The fact of presenting in public, sharing results, and exposing oneself to the gaze of others is experienced simultaneously as tension and opportunity.

The regulation of support remained a relevant condition, although no longer in terms of operational fragility, but rather as sustained pedagogical leadership. The participants appreciated that the accompaniment had not been withdrawn once the innovation was implemented, but continued during the preparation for dissemination and in the subsequent projection. One participant expressed it by saying that there was someone *“always attentive, always, how are you doing? “How are you doing? how are you doing?”* At this point, the support is reinterpreted less as dependence and more as a form of academic and professional care that sustains the continuity of the process.

Intrinsic motivation appears with special strength in this phase. The accounts show that the drive to continue no longer comes solely from institutional obligation or the need to complete a task, but from genuine interest in continuing to develop the innovation, research it, improve it, and share it. A participant pointed out that she now felt *“like doing more,”* continuing to learn and to transfer what she had experienced to other teaching spaces. This reflects a significant shift: learning ceases to be seen as a specific intervention and begins to integrate into the teachers’ own professional horizon.

Finally, personal impact reaches its clearest formulation in this phase. The participants describe the process as growth, transformation, and strengthening of the teaching role. One participant summarized it particularly clearly by stating*: “I, in my case, I would define it with one word: growth… from a professional point of view.”* Other accounts insist that the experience allowed them to rediscover teaching, recognize themselves in community with other colleagues, and expand their willingness to continue innovating and researching. Dissemination, in this sense, does not appear as a simple closure, but as a turning point from which the work carried out acquires density, recognition, and projection. Hence, one of the expressions that best summarizes this phase is the statement that *“this path does not end here. It is just beginning”*.

Overall, the evaluation and dissemination phase show that the closure of the process was not experienced as an ending, but as a rereading, validation, and opening. The participants reinterpreted the effort invested, recognized the learnings that exceeded the initial design of the innovation, and began to envision themselves as part of an academic community with greater cohesion, visibility, and development potential. The experience of spreading it not only legitimized what had been done, but also strengthened the teaching identity, broadened the motivation to continue learning, and consolidated the perception that innovation can also become a path for academic and professional growth.

The foregoing highlights the consolidation of professional identity. Motivation shifts from obligation to genuine interest, and the process is experienced as an opening toward continuous innovation. Furthermore, the implementation of active methodologies is characterized by reduced dependence on technical tools and greater emphasis on factors such as ongoing pedagogical support, self-efficacy, and social recognition.

## 4 Discussion

This section discusses the main results of the study through the identified analytical categories and their development across the six-phase model. The findings show that innovation was experienced by teachers as a progressive process of uncertainty, appropriation, adjustment, support, and professional transformation, rather than as the simple adoption of a methodology. In this way, an integrated reading of the teaching experience during the implementation is proposed, integrating the theoretical perspective of neuroeducation with the findings (Table 4).

**Table 4:** Educational interpretation and neuroeducational lens of the findings.

Main findings	Educational approach	Neuroeducational theoretical perspective
Uncertainty as a persistent component in implementation	Pedagogy of change and educational adaptation (Chen et al., 2025; Magee and Grienberger, 2020) – Uncertainty should be understood as a necessary condition for adaptation to change.	Generation of new neural networks: The progression from initial distrust to self-efficacy generates adaptive patterns that support learning (Ott and Nieder, 2019).
Progressive appropriation of the challenge: from exploration to functional mastery	Transfer of learning to practice (Gautam et al., 2025) – A source of professional growth and capacity development.	Consolidation of neural networks: Repeated activation strengthens and consolidates neural networks (Posner and Rothbart, 2023).
Learning close mentoring (horizontal → pedagogical → mutual)	Continuous and personalized educational support (Liu et al., 2025; Hurskainen et al., 2023; Wang, 2023) – Guidance facilitates decision-making and reduces uncertainty.	Safe environment and well-being: Proximity promotes positive autonomic responses and regulates stress through serotonergic modulation (Colwell et al., 2024). Perceived safety facilitates executive functioning (Kasap et al., 2021).
Continuous adjustment and reconstruction of instructional design (non-linear)	Organizational learning and progressive pedagogical adaptation (Thomas et al., 2025; Perroni et al., 2024) – Maturation requires iterative reorganization based on reflection.	Progressive and cumulative learning: Structured uncertainty dynamics promote deep and sustained transformations (Badripour and Kim, 2026; Murray et al., 2025; Sharpe et al., 2025).
Regulation of support as a critical organizational factor	Sustained pedagogical leadership and institutional support structures (Herodotou et al., 2021; Liu et al., 2025) – Change depends on relationships, roles, and follow-up.	Social component as neurobiological regulator: Socio-emotional mediation modulates autonomic responses and regulates serotonergic stress (Colwell et al., 2024; Michely et al., 2022).
Perceived importance of sharing experiential learning with peers	Mediation of change and organizational learning (Gautam et al., 2025; Perroni et al., 2024) – Sustained transfer occurs through continuity structures.	Support facilitates neural network consolidation: Human interaction promotes neural alignment (Zhu et al., 2022).
Effort–reward balance as a regulator of sustainability	Re-signification of the cost of change and professional validation (Gautam et al., 2025; Perroni et al., 2024; Thomas et al., 2025) – Sustainability depends on how teachers interpret effort.	Reward system co-regulation: Achievement and recognition modulate behavior and persistence through reward-system co-regulation (Fischer and Ullsperger, 2017).
Progressive development of intrinsic motivation	Motivation built from positive pedagogical outcomes (Chen et al., 2025; Thomas et al., 2025) – Motivation strengthens through progressive validation.	Behavioral reinforcement and network consolidation: Progressive achievement enhances motivation via dopaminergic activation (Bech et al., 2023; Deng et al., 2023).
Personal impact: transformation of teaching identity and self-efficacy	Professional identity and capacity for continued innovation (Chen et al., 2025) – Innovation transforms both practices and self-perception.	Optimal neuromodulation: Progressive learning strengthens self-efficacy through consolidation of complex neural networks (Ott and Nieder, 2019).

### 4.1 Uncertainty

Chen et al. (2025) situates preparation for change in understanding, confidence, and attitude toward adopting new methodologies. Along these lines, our findings agree that uncertainty does not disappear, but rather evolves throughout the process: from the initial disorientation in the face of the challenge, to concerns about implementation and transfer, until it becomes academic nervousness and doubts about the scope of the results obtained. In the words of these same authors, uncertainty should not be understood as a barrier, but as a persistent component of teaching innovation processes. In other words, uncertainty is a necessary condition for adapting to change (Magee and Grienberger, 2020). Ultimately, each phase of the model incorporates triggers that promote it, but at the same time integrates factors into the design that modulate it, so that it does not depend solely on the self-regulation of each individual.

### 4.2 Appropriation of the challenge

Findings on the appropriation of the challenge indicate that it evolves from an initial exploration based on trial and error toward functional mastery of the design in real conditions, and culminates in greater confidence to continue learning, communicate the experience, and project it to new contexts. This is consistent with the fact that the transfer of training to practice is perceived by university teachers as a source of professional growth, strengthening of capacities, and greater confidence (Gautam et al., 2025).

### 4.3 Close advice

Our results regarding close advisory not only align with the authors Liu et al. (2025), who emphasize the importance of precise, clear, continuous, and relevant support to the discipline, but these results also present a procedural element: The advisory process is not linear over time, but rather transforms. Initially, it operates as a horizontal accompaniment that facilitates decision-making and reduces the feeling of disorientation. Later, it becomes pedagogical and operational support to organize implementation; and in the final phase, it is redefined as mutual support and academic care during dissemination. Likewise, research with a qualitative design highlights how close communication, ease of access, timely and personalized responses improve the perception of a safe environment and promote well-being during the learning process (Hurskainen et al., 2023; Wang, 2023).

### 4.4 Adjustment and reconstruction

Our findings show that adjustment was not a one-time episode of correction, but a continuous mechanism of design reorganization that evolved in character across the phases of the model. In the preparation and immersion phases, it was primarily aimed at calibrating the load and avoiding overdesign — as participants summarized: *“at first one wants to do everything… overdesign is the same as having no design”* (section 3.1). As the process moved into transfer and implementation, the iterations became more complex: teachers reported successive redesigns to reorganize content, arrange the guiding thread, and maintain coherence between resources, activities, and timing—often under time pressure and with limited room for maneuver (section 3.2). This longitudinal pattern is consistent with studies emphasizing that the implementation of active methodologies requires ongoing pedagogical decisions based on activities, didactic sequences, and student participation (Thomas et al., 2025), and that sustained pedagogical change depends on organizational learning, resource creation, and processes of progressive adaptation over time (Perroni et al., 2024). Taken together, these findings suggest that the reconstruction of instructional design should not be understood as a sign of weakness, but as a condition inherent to its pedagogical maturation.

### 4.5 Regulation of support

The studies conducted by Herodotou et al. (2021) and Liu et al. (2025) show that the use and maintenance of an innovation depend not only on the individual teacher’s willingness, but also on support structures, monitoring, clarity, and institutional relevance. Our results reinforce and extend this finding. In the transfer and implementation phase, progress was critically conditioned by dependencies on specific roles and technical access conditions: in one particularly illustrative case, the implementation at a given site was interrupted when the responsible teacher went on medical leave, because the platform resource “was lost because they could not log in” (section 3.2). Participants explicitly identified this structural vulnerability, suggesting that project coordinators should have access to all platform fields “precisely so as not to depend on a single account or person” (section 3.2). Over time, these operational dependencies were redefined as the need for sustained pedagogical leadership capable of ensuring continuity. In this sense, the regulation of support makes clear that pedagogical and disciplinary change does not occur solely at the individual level, but within an organizational system that enables, limits, or interrupts its development (Herodotou et al., 2021; Liu et al., 2025), underscoring the social and structural components as key regulatory factors (Colwell et al., 2024; Michely et al., 2022).

### 4.6 Teacher transfer

Regarding teacher transfer, Gautam et al. (2025) links it to professional growth, confidence, and development, while Perroni et al. (2024) ground it in a pedagogical approach that depends on structures that ensure continuity, shared understanding, and organizational learning. Our findings converge with both perspectives while adding a key nuance: teacher transfer gains value when supported by monitoring and continuity structures, though its adoption remains uneven among teachers who did not participate in the initial process. In the dissemination phase, transfer extends beyond individual practice to reach other teams, faculties, and cohorts, functioning not as mere technical replication, but as a process of mediation, support, and negotiated change (Gautam et al., 2025; Perroni et al., 2024). Scaffolding strategies, advisory support, and tools reinforce perceived safety and further facilitate this process (Koudsia and Kirchner, 2024).

### 4.7 Effort-reward balance and the development of intrinsic motivation

The sustainability of the implementation was shaped, above all, by how teachers re-signified the cost of engaging in it across the different phases (Perroni et al., 2024; Thomas et al., 2025). In the initial phases, effort was experienced as valuable intensity—demanding, but productive and meaningful. During transfer and implementation, it became a structural tension linked to time, workload, and contractual conditions: one participant stated that time was *“by far the biggest obstacle,”* and for adjunct teachers, the absence of monetary compensation meant that “part of the collaboration ended up being more instrumental than truly shared” (section 3.2). However, when the instructional design began to show coherence and yield visible results, the effort was consistently and positively reinterpreted—experienced not as wear and tear but as a different kind of fatigue, linked to learning and achievement. This trajectory aligns with the idea that the sustainability of innovation depends on the way teachers re-signify the cost of engaging in it, and that this re-signification is enabled by the progressive perception of pedagogical meaning and institutional recognition (Gautam et al., 2025; Perroni et al., 2024; Thomas et al., 2025).

From this reinterpretation of effort, a deepening of intrinsic motivation emerged. Participants described a shift from compliance-driven engagement to genuine interest in continuing to develop, improve, and share the innovation—as one participant noted, she now felt “like doing more*…* continuing to learn and sharing it” (section 3.3). This is consistent with evidence that motivation does not operate as a prior trait of the teacher, but as a disposition that is strengthened through the experience of successful implementation and the progressive validation of the work carried out (Chen et al., 2025; Thomas et al., 2025). The progressive achievement of concrete results and the awareness of pedagogical change reinforce behavior and sustain intrinsic motivation through mechanisms theoretically aligned with dopaminergic activation and reward prediction (Bech et al., 2023; Deng et al., 2023).

### 4.8 Personal impact

Beyond motivational change, the findings document a broader transformation in the teachers’ relationship with their own professional identity. Participants described the process as one of growth, rekindled engagement with teaching, and expanded self-efficacy: “I, in my case, I would define it with one word: growth… from a professional point of view” (section 3.3). This aligns with Chen et al. (2025) argument that change processes involve not only new practices but a transformation in how teachers perceive themselves as professionals capable of continuing to innovate. Importantly, this impact was not limited to individuals: participants reported recognizing themselves as part of a broader academic community, which reinforced their willingness to continue researching and sharing their work. In this sense, personal impact represents the integrating outcome of the six-phase process—the point at which the experiential, motivational, and relational dimensions of the implementation converge into a transformed professional self-perception.

Across the six phases, the findings reveal a process of progressive professional development in which uncertainty, support, effort, motivation, and personal impact do not operate as isolated variables but as interconnected elements that evolve over time. A key contribution of the model lies in its temporal dimension: time functions not merely as a container for activities, but as a structural design variable that enables the cumulative development of successive cycles of imbalance and adaptation—what Amemasor et al. (2025) associate with the conditions for deep learning. Activities within the model are deliberately sequenced to sustain motivation, foster reflection, and enhance adaptive capacity amid persistent uncertainty over a six-month implementation period. This temporal articulation is precisely what distinguishes the present model from single-session or phase-agnostic neuroeducational frameworks.

Our six-phase model differs from existing frameworks by explicitly acknowledging the limits of neuroscientific application in educational contexts. While Pradeep et al. (2024) report specific neural mechanisms, and Rodríguez Dueñas et al. (2026) outline approaches for integrating brain-based findings into instructional design, we adopt a more cautious stance. The processes identified in this study—uncertainty, mentoring, intrinsic motivation, and effort-reward balance—are derived from qualitative analysis and should not be interpreted as having direct neurobiological grounding. Our intention is to document observable teaching processes, to draw on neuroeducational literature as a theoretical reference point, and to avoid the speculative generalizations identified in other frameworks (Boysen, 2024; Pastor et al., 2022).

## 5 Study limitations

This study presents some limitations that must be considered when interpreting its findings. First, the study worked with a limited sample of 10 teachers belonging to a single institutional context and to a specific innovation of the Faculty of Medicine, so the results should be understood as situated and exploratory in nature, rather than broadly generalizable. Second, the qualitative process evaluation design allows for understanding experiences, meanings, and implementation conditions, but not for establishing causal relationships or attributing effects of the model to external variables. Third, although the study relies on a neuroscientific framework, it did not incorporate direct neurobiological measurements nor did it evaluate student learning outcomes; therefore, the relationship with dopaminergic and serotonergic co-regulation should be understood on a theoretical-interpretive level. Likewise, the evidence was based mainly on the participants’ accounts, so the findings depend on the way in which they reconstructed and interpreted their experience when communicating it. Finally, the proximity of part of the research team to the design and implementation of the model, even when addressed through systematic reflexivity, also constitutes a potential source of interpretive partiality.

## Data Availability

The raw data supporting the conclusions of this article will be made available by the authors, without undue reservation.

## References

[ref1] Amemasor S. K. Oppong S. O. Ghansah B. Benuwa B. B. Essel D. D. (2025). A systematic review on the impact of teacher professional development on digital instructional integration and teaching practices. Front. Educ. 10:1541031. doi: 10.3389/feduc.2025.1541031

[ref2] Antón-Sancho Á. Vergara D. Fernández-Arias P. Zhao J. (2022). Impact of the COVID-19 pandemic on the use of ICT tools in science and technology education. J. Technol. Sci. Educ. 12:1860. doi: 10.3926/jotse.1461

[ref4] Badripour A. Kim T. (2026). Causal links between serotonin dynamics and cued fear learning: evidence from experimental studies. Front. Comput. Neurosci. 20, 1–14. doi: 10.3389/fncom.2026.1750926, 41657916 PMC12876228

[ref5] Baraldi V. (2021). John Dewey: La educación como proceso de reconstrucción de experiencias. Revista de la Escuela de Ciencias de la Educación 1, 68–76. doi: 10.35305/rece.v1i16.587

[ref7] Bech P. Crochet S. Dard R. Ghaderi P. Liu Y. (2023). Striatal dopamine signals and reward learning. Function 4:zqad056. doi: 10.1093/function/zqad056, 37841525 PMC10572094

[ref8] Boysen G. A. (2024). A critical analysis of the research evidence behind CAST's universal design for learning guidelines.Policy futures. Education 22, 1219–1238. doi: 10.1177/14782103241255428

[ref9] Buckley A. N. (2021). A multiple Goals Perspective on Burnout Disclosure and Support among Attending Physicians. Lexington: University of Kentucky.

[ref4007] Cando R. V. Q. Idrovo M. N. I. Carranza S. L. C. Espinoza J. F. J. (2025). La emoción como motor del aprendizaje: estrategias didácticas que desafían la mente y conectan con la experiencia. Star. Sci. Multidiscip. J. 2, 1–15., 24979285

[ref4006] Chen J. Du X. Bandi S. de Carvalho Guerra A. O. P. Hansen S. Joshi S. (2025). Exploring university educators’ readiness for changing to PBL: a case from India. Int. J. Acad. Dev. 1–17. doi: 10.1080/1360144X.2025.2574982

[ref11] Coddington L. T. Lindo S. E. Dudman J. T. (2023). Mesolimbic dopamine adapts the rate of learning from action. Nature 614, 294–302. doi: 10.1038/s41586-022-05614-z, 36653450 PMC9908546

[ref12] Colwell M. J. Tagomori H. Shang F. Cheng H. I. Wigg C. E. Browning M. . (2024). Direct serotonin release in humans shapes aversive learning and inhibition. Nat. Commun. 15:6617. doi: 10.1038/s41467-024-50394-x, 39122687 PMC11315928

[ref13] Courtiol E. Menezes E. C. Teixeira C. M. (2021). Serotonergic regulation of the dopaminergic system: implications for reward-related functions. Neurosci. Biobehav. Rev. 128, 282–293. doi: 10.1016/j.neubiorev.2021.06.022, 34139249 PMC8335358

[ref14] Crous G. Rodríguez-Rodríguez J. Padilla-Petry P. (2024). Metodologías activas en la educación superior: El caso de la docencia no-presencial durante la pandemia de la COVID-19. Educatio Siglo XXI 42, 9–32. doi: 10.6018/educatio.550001

[ref15] Dahal N. D. N. (2025). Qualitative data analysis: reflections, procedures, and some points for consideration. Front. Res. Metrics Anal. 10, 1–10. doi: 10.3389/frma.2025.1669578, 41098525 PMC12518266

[ref16] Deng Y. Song D. Ni J. Qing H. Quan Z. (2023). Reward prediction error in learning-related behaviors. Front. Neurosci. 17, 1–10. doi: 10.3389/fnins.2023.1171612, 37662112 PMC10471312

[ref18] Elouafi L. Lotfi S. Talbi M. (2021). Progress report in neuroscience and education: experiment of four neuropedagogical methods. Educ. Sci. 11:373. doi: 10.3390/educsci11080373

[ref19] Evans P. Vansteenkiste M. Parker P. Kingsford-Smith A. Zhou S. (2024). Cognitive load theory and its relationships with motivation: a self-determination theory perspective. Educ. Psychol. Rev. 36, 1–25. doi: 10.1007/s10648-023-09841-2, 30311153

[ref20] Fischer A. G. Ullsperger M. (2017). An update on the role of serotonin and dopamine in error processing and cognitive control. Front. Hum. Neurosci. 11, 1–10. doi: 10.3389/fnhum.2017.00484, 29075184 PMC5641585

[ref21] Fredkove W. Mann E. Abudiab S. De Acosta D. Garcia Y. Hoffman S. J. . (2024). Utilizing rapid qualitative assessment and thematic analysis methods to identify and share promising case investigation and contact tracing practices with people in refugee, immigrant, and migrant communities during COVID-19. Front. Public Health 12:1359145. doi: 10.3389/fpubh.2024.1359145, 39022416 PMC11252043

[ref4005] Gautam A. Dahal N. Hasan M. K. (2025). University faculty perceptions of training transfer and professional growth: a qualitative study. Front. Educ. 10:1553377. doi: 10.3389/feduc.2025.1553377

[ref22] Herd S. Krueger K. Nair A. Mollick J. O’Reilly R. (2021). Neural mechanisms of human decision-making. Cogn. Affect. Behav. Neurosci. 21, 35–57. doi: 10.3758/s13415-020-00842-0, 33409958

[ref23] Herodotou C. Maguire C. McDowell N. Hlosta M. Boroowa A. (2021). The engagement of university teachers with predictive learning analytics. Comput. Educ. 173:104285. doi: 10.1016/j.compedu.2021.104285

[ref24] Hill T. Dao M. (2021). Personal pasts become academic presents: engaging reflexivity and considering dual insider/outsider roles in physical cultural fieldwork. Qual. Res. Sport, Exerc. Health 13, 521–535. doi: 10.1080/2159676X.2020.1731576

[ref25] Hoff C. H. C. Strømme H. S. (2025). The unbearable lightness of laughing: a reflexive thematic analysis of smiles and laughter in five psychotherapy training processes. Front. Psychol. 16:1720110. doi: 10.3389/fpsyg.2025.1720110, 41341729 PMC12671044

[ref27] Hurskainen J. Wenström S. Uusiautti S. (2023). A PRIDE-theory-based analysis of a positive learning environment in a Finnish vocational education and training (VET). Institutions 13, 74–99. doi: 10.3384/njvet.2242-458x.2313274

[ref4004] Kasap E. Z. Ağzıtemiz F. Ünal G. (2021). Cognitive, mental and social benefits of interacting with nature: a systematic review. J. Happiness Health 1, 16–27. Available online at: https://journalofhappinessandhealth.com/index.php/johah/article/view/1

[ref30] Kikuchi Y. Noriuchi M. (2020). “The romantic brain: secure attachment activates the brainstem centers of well-being,” in The Romantic Brain, ed. Noriuchi M. (Cham: Springer), 135–147.

[ref31] Kok A. (2022). Cognitive control, motivation and fatigue: A cognitive neuroscience perspective. Brain Cogn. 160:105880. doi: 10.1016/j.bandc.2022.105880, 35617813

[ref32] Koudsia S. Kirchner M. (2024). Reducing cognitive overload for students in higher education: a course design case study. J. Higher Educ. Theory Practice 24, 97–124. doi: 10.33423/jhetp.v24i10.7382

[ref34] Li Q. Ochiai H. Ochiai T. Takayama N. Kumeda S. Miura T. . (2022). Effects of forest bathing (shinrin-yoku) on serotonin in serum, depressive symptoms and subjective sleep quality in middle-aged males. Environ. Health Prev. Med. 27:44. doi: 10.1265/ehpm.22-00136, 36328588 PMC9665960

[ref35] Liebana S. Laffere A. Toschi C. Schilling L. Moretti J. Podlaski J. . (2025). Dopamine encodes deep network teaching signals for individual learning trajectories. Cell 188, 3789–3805.e33. doi: 10.1016/j.cell.2025.05.025, 40505657 PMC7619352

[ref36] Liu Z. Lin R. Luo M. (2020). Reward contributions to serotonergic functions. Annu. Rev. Neurosci. 43, 141–162. doi: 10.1146/annurev-neuro-093019-112252 32640931

[ref4003] Liu Z. Sulaiman T. Che Nawi N. R. (2025). Between development and surveillance: faculty perceptions and challenges of instructional coaching in an underdeveloped university context. Front. Educ. 10:1637546. doi: 10.3389/fpubh.2023.1199571, 37427273 PMC10328089

[ref37] López V. Carrasco-Aguilar C. Jervis P. Torres-Vallejos J. Ramírez M. T. González J. P. Á. . (2025). Qualitative evaluation of the feasibility of a national whole-school program for reducing school violence and improving school climate in Chile. Front. Educ. 10:1471235. doi: 10.3389/feduc.2025.1637546

[ref38] Luria E. Shalom M. Levy D. A. (2021). Cognitive neuroscience perspectives on motivation and learning: revisiting self-determination theory. Mind Brain Educ. 15, 5–17. doi: 10.1111/mbe.12275

[ref39] Magee J. C. Grienberger C. (2020). Synaptic plasticity forms and functions. Annu. Rev. Neurosci. 43, 95–117. doi: 10.1146/annurev-neuro-090919-022842, 32075520

[ref41] Michely J. Eldar E. Erdman A. Martin I. Dolan R. (2022). Serotonin modulates asymmetric learning from reward and punishment in healthy human volunteers. Commun. Biology 5:812. doi: 10.1038/s42003-022-03690-5, 35962142 PMC9374781

[ref42] Moore G. F. Audrey S. Barker M. Bond L. Bonell C. Hardeman W. . (2015). Process evaluation of complex interventions: Medical Research Council guidance. BMJ 350:h1258. doi: 10.1136/bmj.h1258, 25791983 PMC4366184

[ref43] Murray E. Horner A. J. Göbel S. M. (2025). A meta-analytic review of the effectiveness of spacing and retrieval practice for mathematics learning. Educ. Psychol. Rev. 37:75. doi: 10.1007/s10648-025-10035-1

[ref44] O’Brien B. C. Harris I. B. Beckman T. J. Reed D. A. Cook D. A. (2014). Standards for reporting qualitative research: a synthesis of recommendations. Acad. Med. 89, 1245–1251. doi: 10.1097/ACM.0000000000000388, 24979285

[ref45] O’Connor C. Joffe H. (2020). Intercoder reliability in qualitative research: debates and practical guidelines. Int J Qual Methods 19, 1–13. doi: 10.1177/1609406919899220

[ref46] Oettl L. L. Scheller M. Filosa C. Wieland S. Haag F. Loeb C. . (2020). Phasic dopamine reinforces distinct striatal stimulus encoding in the olfactory tubercle driving dopaminergic reward prediction. Nat. Commun. 11:3460. doi: 10.1038/s41467-020-17257-7, 32651365 PMC7351739

[ref47] Oliveira G. Grenha Teixeira J. Torres A. Morais C. (2021). An exploratory study on the emergency remote teaching experience of higher education students and teachers during the COVID-19 pandemic. Br. J. Educ. Technol. 52, 1357–1376. doi: 10.1111/bjet.13112, 34219758 PMC8237053

[ref49] Ott T. Nieder A. (2019). Dopamine and cognitive control in prefrontal cortex. Trends Cogn. Sci. 23, 213–234. doi: 10.1016/j.tics.2018.12.006 30711326

[ref50] Pastor C. A. Martínez-Martín I. Caparrós-Martín E. Galindo-Domínguez H. Silva-Laguardia M. M. Hernández-Portero G. . (2022). Enseñar pensando en todos los estudiantes: El modelo de Diseño Universal para el Aprendizaje (DUA), vol. 50 Madrid: Ediciones SM.

[ref4002] Perroni N. S. (2024). How can we change if we are the same people?: Chilean academic leaders’ experiences implementing a practice-based teacher education approach. Teach. Teach. Educ. 138:104412. doi: 10.1016/j.tate.2023.104412, 38090170

[ref51] Posner M. I. Rothbart M. K. (2023). How understanding and strengthening brain networks can contribute to elementary education. Front. Public Health 11:1199571. doi: 10.3389/fpubh.2023.1199571, 37427273 PMC10328089

[ref52] Pradeep K. Sulur Anbalagan R. Thangavelu A. P. Aswathy S. Jisha V. G. Vaisakhi V. S. (2024). Neuroeducation: understanding neural dynamics in learning and teaching. Front. Educ. 9:1437418. doi: 10.3389/feduc.2024.1437418

[ref54] Ribeiro-Silva E. Amorim C. Aparicio-Herguedas J. L. Batista P. (2022). Trends of active learning in higher education and students’ well-being: a literature review. Front. Psychol. 13:844236. doi: 10.3389/fpsyg.2022.844236 35519651 PMC9062227

[ref55] Rodríguez Dueñas J. S. Vargas Sánchez A. D. Boude Figueredo O. Almenarez Mendoza F. (2026). Charting the growth of brain-based learning in higher education instructional design. Front. Educ. 11:1677395. doi: 10.3389/feduc.2026.1677395

[ref56] Sharpe B. T. Trotter M. G. Hale B. J. (2025). Sustaining student concentration: the effectiveness of micro-breaks in a classroom setting. Front. Psychol. 16:1589411. doi: 10.3389/fpsyg.2025.1589411, 40851619 PMC12369656

[ref57] Skivington K. Matthews L. Simpson S. A. Craig P. Baird J. Blazeby J. M. . (2021). A new framework for developing and evaluating complex interventions: update of Medical Research Council guidance. BMJ 374:n2061. doi: 10.1136/bmj.n2061, 34593508 PMC8482308

[ref59] Smith D. D. Bartley J. E. Peraza J. A. Bottenhorn K. L. Nomi J. S. Uddin L. Q. . (2025). Dynamic reconfiguration of brain coactivation states associated with active and lecture-based learning of university physics. NPJ Sci. Learn. 10:55. doi: 10.1038/s41539-025-00348-9, 40819140 PMC12357887

[ref4001] Thomas M. B. Muscat A. Zuccolo A. Nascimento C. Watt A. (2025). Navigating Pedagogical Innovation in Higher Education: Education Academics’ Experiences with Active and Inquiry-Based Learning in Intensive Teaching. Innov. High. Educ. 50, 1917–1943. doi: 10.1007/s10755-025-09807-y, 24979285

[ref62] Tong A. Sainsbury P. Craig J. (2007). Consolidated criteria for reporting qualitative research (COREQ): a 32-item checklist for interviews and focus groups. Int. J. Qual. Health Care 19, 349–357. doi: 10.1093/intqhc/mzm042, 17872937

[ref64] Wang T. (2023). Teachers as the agent of change for student mental health: the role of teacher care and teacher support in Chinese students’ well-being. Front. Psychol. 14:1283515. doi: 10.3389/fpsyg.2023.1283515, 38090170 PMC10713756

[ref66] Wu C. Zhang W. Hu L. Li M. (2026). Research on middle school teachers’ technostress empowered by artificial intelligence. Front. Artificial Intell. 8:1732088. doi: 10.3389/frai.2025.1732088, 41626577 PMC12852380

[ref67] Zhu Y. Leong V. Hou Y. Zhang D. Pan Y. Hu Y. (2022). Instructor–learner neural synchronization during elaborated feedback predicts learning transfer. J. Educ. Psychol. 114, 1427–1441. doi: 10.1037/edu0000707

